# Transition Probabilities of Forbidden Lines

**DOI:** 10.6028/jres.068A.004

**Published:** 1964-02-01

**Authors:** R. H. Garstang

## Abstract

This paper describes calculations of the transition probabilities of forbidden lines (magnetic dipole and electric quadrupole radiation) of laboratory and astrophysical interest. Results are given for Ti III, Cr II, Cr IV, Mn V, Mn VI, Fe VI, Fe VII, Ni I, Cu II, Ga I, Ge I, Ge II, As I, As III, Se I, Br I, Br II, Kr II, Kr III, Rb III, In I, Sn I, Sn II, Sb I, Sb III, Te I, I I, I II, Xe II, Xe III, Cs III, Hg II, Tl I, Pb I, Pb II, Bi I, Bi II, Bi III, Po I, and Rn II.

## 1. Introduction

This paper presents the results of calculations of the transition probabilities of forbidden lines for a number of atoms and ions of astrophysical or laboratory interest. Much work has been done in the past on atoms in the first two short periods and their isoelectronic sequences, and selected ions in the iron group have also been studied. A few additional ions in the iron group need study, chiefly with a view to astrophysical applications. These form the subject of sections 2 through 4 of the present paper. There have been few calculations on forbidden lines of heavier elements. Only a few are of possible astrophysical interest; a number have been observed in the laboratory. Calculations of their transition probabilities are not difficult, and have been carried out for many atoms and ions, the results being given in section 5 of this paper. They include, it is thought, every case in which one or more forbidden lines in an atom has been observed in laboratory sources, and for which transition probabilities have not previously been computed.

A comprehensive survey of this subject has recently been given [1],^2^ and in this paper we shall only give such details as are immediately relevant to the individual atoms being considered. Unless otherwise mentioned all observed atomic energy levels have been taken from [2]. The procedure, now well established, is to take the quantum mechanical energy matrices including spin-orbit interaction (and, where necessary, configuration interaction) and determine the parameters (radial integrals) in these matrices so that the eigenvalues of the matrices reproduce the observed energies as accurately as possible. The eigenvectors provide the transformation from *LS*-coupling to intermediate coupling. The matrices of the square roots of line strengths are set up, the transformation to intermediate coupling carried out, and the final line strengths converted to transition probabilities. The total transition probability for a line is the sum of the magnetic dipole transition probability (*A_m_*) and the electric quadrupole transition probability (*A_q_*).

## 2. Lines of Ti III, Mn VI, Fe VII, Cr IV_;_ Mn V, and Fe VI

In Ti III, Mn VI, and Fe VII the transitions take place within the 3*d^2^* configuration. Fe VII was studied by Pasternack [3]; the other two ions do not appear to have been the subject of earlier forbidden line calculations. The spin-orbit matrix was taken from Condon and Shortley [4, p. 269]; the electrostatic energies were treated as arbitrary parameters. The parameters obtained by fitting the theory to the observed energies are given in table 1; E(^1^S) was estimated using the theoretical formulas for electrostatic energies [5] including an *αL(L+*1) correction [6]. The resulting calculated energies are given in table 2. The only significant comparison with observation is for the ^3^F and ^3^P term intervals, for which the agreement is quite good, showing that the intermediate coupling theory provides a fair representation of the atomic fine structure. The line strength matrix for magnetic dipole radiation was obtained from formulae of Shortley [7], the electric quadrupole line strength matrix from Pasternack [3] with corrections by Garstang [8], and the radial integrals

$$s_{q}=\int_{0}^{\infty }r^{2}P^{2}(3d)dr$$

were obtained from wave functions given by Watson [9]. The values of *s_q_* used are listed in table 1. The intermediate coupling transformations and the final transition probabilities were computed in the usual way. The results are listed in table 3. *A_m_* and *A_q_* denote spontaneous emission transition probabilities in sec^−1^; the total transition probability is *A_m_+A_q_.*

The results obtained for Mn VI may be compared with those obtained by Pasternack for Fe VII. The results show the usual trend of increasing transition probabilities along the isoelectronic sequence. Pasternack used *s_q_*= 1.52 for Fe VII, based on a rather crude estimate of certain screening constants. A better estimate of *s_q_* can now be obtained from Watson’s wave functions, based on an extrapolation of *s_q_*^−½^ along the isoelectronic sequence, Mn VI being the highest ion of this sequence for which wave functions were computed. The resulting estimate of *s_q_* is given in table 4. The magnetic dipole transition probabilities obtained by Pasternack are unchanged on a change of *s_q_.* The electric quadrupole strengths must be multiplied by the square of the ratio of the new *s_q_* to the old *s_q_.* The electric quadrupole transition probabilities must be multiplied by this same correction factor, which is given in table 4. Pasternack also performed calculations on Cr IV, Mn V, and Fe VI, for which the transitions take place within the 3*d^s^* configuration. We have computed improved values of *s_q_* for these three ions from the wave functions published by Watson. The values of *s_q_* are given in table 4 together with correcting factors by which Pasternack’s electric quadrupole transition probabilities are to be multiplied to take account of the revised *s_q_.* As for Fe VII, the magnetic dipole transition probabilities are unaffected by the change in *s_q_.*

Bowen [10] observed the transitions ^3^F_2_–^1^D_2_ (λ6518.3), ^3^F_4_–^3^P_2_(λ5894.0), and ^3^F_3_–^3^P_1_(λ5776.4) of Mn VI in a planetary nebula. No forbidden lines of Ti III appear to have been observed.

## 3. Lines of Cr II

A complete study of the forbidden lines of Cr II would necessitate a very long investigation which the astronomical importance of this ion is hardly sufficient to justify. It so happens that the one multiplet of most importance can be simply treated. This is the transition 3*d^5^* ^6^S–3*d*^4^4*s* ^6^D. The four strongest lines of this multiplet were observed by Thackeray [11] in the infrared spectrum of *η* Carinae. ^6^S and ^6^D are the only sextet terms in their respective configurations, and consequently there are no large off-diagonal matrix elements connecting them with other terms. Experience (especially with Fe II [12]) has shown that in these circumstances, for a transition allowed in *LS*-coupling, magnetic dipole radiation is likely to be negligible and the *LS*-coupling approximation will give excellent values for the electric quadrupole transition probabilities. The total multiplet strength is given in the appendix to the present paper. The individual line strengths were obtained, and converted to transition probabilities. The radial integral

$$S_{q}=\int_{0}^{\infty }r^{2}P(3d)P(4s)dr$$

was estimated by extrapolating *s_q_*^−½^ from the values previously obtained for Fe II [12] and Ni II [13]. We adopted *s_q_*= −3.5. The results are listed in table 5.

## 4. Lines of Ni I and Cu II

In connection with some intensity calculations on permitted transitions the writer undertook a study of Ni I, and part of that work may also be used in calculations on forbidden lines. The lower levels of Ni I arise from the configurations 3*d*^10^, 3*d*^9^4*s*, and *Sd*^8^4*s*^2^. The energy levels have been fitted to the theory and parameters estimated in the usual way. The formulae for the electrostatic energies were obtained from [4, p. 299] and [5, eq (78)], an *aL*(*L+*1) correction [6] being added for the 3*d^8^*4*s^2^* configuration. The configuration interaction matrix elements were obtained from [14, eqs (75) and (81)]. The spin-orbit interaction matrix elements were taken from [4, p. 269] with changes in the sign of *ζ* (for *d^8^* or *d*^9^ instead of *d^2^* or *d*), and of the phase of *ds* (instead of *sd*). The adopted parameters are given in table 6. The configuration interaction parameter H_2_ was neglected; a study by Racah and Shadmi [15] showed that H_2_ is very small for Ni II, and presumably also for Ni I. Table 7 gives the observed energies and those calculated from the parameters in table 6. The Landé *g*-factors are also given. The overall agreement of observation and calculation is very satisfactory. The line strength matrices were obtained in the usual way [3, 7, 8, 16] and intermediate coupling transformations carried out to obtain the final line strengths and transition probabilities. The only problem which arose was the adoption of numerical values of the radial integrals for electric quadrupole radiation. These were obtained by extrapolation of results for various stages of ionization of manganese, iron, and nickel which had been obtained in earlier work. The adopted radial integrals are listed in table 6 and the transition probabilities in table 8.

In Cu II we are only interested in the 3*d*^10^ and 3*d*^9^4*s* configurations, but the 3*d*^8^4*s*^2^ configuration must be included in the calculations. Cu II has been studied by Racah and Shadmi [15], and we have adopted the numerical values of the parameters which they obtained. The Ni I work was followed so far as necessary for Cu II. Examination showed that there are no magnetic dipole transitions between 3*d*^10^ and 3*d*^9^4*s*. For electric quadrupole radiation ^1^S_0_–^3^D_1_ and ^1^S_0_–^3^D_3_ are strictly forbidden. The remaining transitions were computed in the same way as for Ni I. The radial integrals for electric quadrupole radiation were estimated by extrapolation of s*_q_*^−½^ from Fe II [12] and Ni II [13]. We adopted *s_q_*(*d*^10^–*d*^9^*s*) = −2.0, *s_g_(d^9^s–d^8^s^2^*) = −1.5. s*_q_*(*d*^9^*s–d*^9^*s*) = +1.0. The resulting transition probabilities are given in table 9.

The transition probabilities of Cu II are of interest in connection with observations of the spectrum of *η* Carinae by Thackeray [17]. He observed the transition 3*d*^10 1^S_0_–3*d*^9^4*s* ^1^D_2_ at λ3806. Our results show that this line is indeed the strongest forbidden line of Cu II, and explain the absence of the line. The ^1^S_0_−^3^D_2_ line might be observable under suitable conditions; but in *η* Carinae the relatively high densities of the gas (in comparison with planetary nebulae) would enhance the importance of collisional excitation and de-excitation relative to radiative processes, and the ^1^S_0_–^3^D_2_ line would then be weaker than the ^1^S_0_–^1^D_2_ line by an order of magnitude because of its lower transition probability.

## 5. Forbidden Lines in Heavier Atoms (*Z*>29)

Transition probabilities are available for only a very few forbidden lines of atoms with *Z*>29. The remainder of this paper will be devoted to presenting some additional data on such transitions. The ^2^D–^2^S doublets in Rb I and Cs I have been discussed in detail [4, p. 256] and will not be further considered here. Results have previously been obtained also for Pb I [18], Br II [19], I II [20], Kr III [21], Rn II [22], Xe II [22], and Xe III [22].

### 5.1. *np* and *np*^5^ Configurations

The only observation of the ^2^P_3/2_–^2^P_½_ transition within an *np* configuration appears to be that in Pb **II** 6*p* at λ7099.8, first observed by Walters [23], and identified and studied by Cole [24]. The ^2^P_½_^–2^P_3/2_ transition in the *np*^5^ configuration has been observed in four cases: Xe II 5*p^5^* at λ94N7.8 and Rn II 6*p^b^* at λ3235.8 by Edlén [22], I I 5p^5^ at λ13149 by Eshbach and Fisher [25] and Iviess and Corliss [26], and Br I 4*p*^5^ at λ27130 by J. C. Polanyi [private communication].

Transition probabilities have been calculated from the *LS*-coupling formulae for magnetic dipole line strengths given by Shortley [7] and for electric quadrupole line strengths by Garstang [8, eqs (3), (5), (6) and par. 5]. It is found that for *np* and *np^5^* configurations

$$\begin{array}{cc} S_{m}=\frac{4}{3} & S_{q}=\frac{10}{3}s_{q}^{2} \end{array}$$

where

$$s_{q}=\frac{2}{5}\int_{0}^{\infty }r^{2}P^{2}(np)dr$$

and the factor (2/5) has been included in *s_q_* for *p* electrons (cf *s_q_* for *p* electrons) in accordance with custom [27]. P(*np*) is the radial wave function for an *np* electron. The transition probabilities obtained are listed in tables 10 and 11, where we have included for completeness the two values calculated by Edlén [22]. The estimation of *s_q_* is discussed below.

### 5.2. *np*^2^, *np*^3^ and *np*^4^ Configurations

Observations of the forbidden line ^1^S_0_–^3^P_1_ in Pb I 6*p*^2^ at λ4618 played an important part in the history of the subject, for it was by means of observations of the Zeeman effect on this line that Niewodniczanski [28] first demonstrated the occurrence of magnetic dipole radiation. The line ^1^D_2_–^3^P_1_ (λ7330) was first observed by Walters [23], λ4618 by Gieseler and Grotrian [29], and by Sur [30], and ^1^D_2_–^3^P_0_ (λ4659) and ^1^S_0_–^3^P_2_ (λ5313) by Niewodniczanski [31], who also studied λ4618 and λ7330. Mrozowski [32] found ^1^D_2_–^3^P_2_ (λ9250). Mrozowski studied the relative intensities *I*(4618): *I*(5313) and *I*(4659): *I*(7330): *I*(9250). The Zeeman effect of λ4618, λ4659, λ5313, and λ7330 was studied by Jenkins and Mrozowski [33]. Gerjuoy [18] gave a detailed discussion of the intensities, to be referred to again below.

Forbidden lines of Bi II 6*p^2^* were observed by Cole and Mrozowski [34], and Cole [35], who obtained ^1^S_0_–^3^P_1_ (λ3241), ^1^S_0_–^3^P_2_(λ3683), ^1^D_2_–^3^P_1_(λ4850), ^1^D_2_–^3^P_2_(λ5914), and ^3^P_1_–^3^P_0_(λ7503), and studied their hyperfine structure.

Forbidden lines of As I 4*p^3^* ^2^P_3/2_–^4^S_3_/_2_(λ5362) and ^2^P_½_–^4^S_3/2_(λ5498) and of Sb I 5*p*^3 2^P_3/2_–^4^S_3/2_(λ5415) and ^2^P_½_–^4^S_3/2_(λ6098) were observed by Hults and Mrozowski [36]. In Bi I 6*p^s^* Toshniwal [37] found ^2^D_3/2_–^4^S_3/2_(λ8755), ^2^D_5/2_–^4^S_3/2_(λ6476), and ^2^P_½_–^4^S_3/2_ (λ4615), and Mrozowski [38] made a study of these lines together with ^2^P_3/2_–^4^S_3/2_(λ3014), ^2^P_3/2_–^2^D_3/2_ (λ4597), and ^2^P_3/2_–^2^D_5/2_(λ5640) which he found. Mrozowski made hyperfine structure observations which proved that all these Bi I transitions are predominantly magnetic dipole radiation. For λ6476, the best resolved line, electric quadrupole radiation was less than 0.15 of the total. Mrozowski also pointed out the need for more laboratory intensity measurements on the forbidden lines of Bi I and other atoms.

Ruedy and Gibbs [39] found the lines ^1^S_0_–^1^D_2_ (λ7768) and ^1^S_0_–^3^P_1_ (λ4887) in Se I 4*p*^4^. Niewod- niczanski and Lipinski [40] found ^1^S_0_–^1^D_2_ (λ7909), ^1^S_0_–^3^P_1_ (λ5420), and ^1^S_0_–^3^P_2_ (λ4309) in Te I 5*p*^4^ λ5420 being much stronger than the others. Mrozowski [41] observed ^1^S_0_–^3^Pi (λ3862), ^1^D_2_-^3^P_2_ (λ4611), and ^3^P_1_–^3^P_2_ (λ5940) in Po I 6*p*^4^. Martin and Tech [19] found ^1^S_0_–^3^P_1_ (λ4042) and ^1^D_2_–^3^P_2_ (λ8270) in Br II 4*p*^4^. Martin and Corliss [20] found ^1^S_0_**–**^3^P_1_ (λ4460) and ^1^D_2_–^3^P_2_ (λ7283) in I II *5p*^4^ and Edlén [22] found ^1^D_2_–^3^P_2_ (λ5846.3) and ^3^P_1_–^3^P_2_ (λ10206.5) in Xem 5*p*^4^. Kr III ^1^D_2_-^3^P_2_ (λ6827) may be present in spectra of RS Ophiuchi [54], but later work cast considerable doubt on the identification [61].

General formulae for line strengths and transition probabilities in *p*^2^, *p*^3^, and *p*^4^ configurations were given by Shortley, Aller, Baker, and Menzel [27].

$$\begin{array}{l} \Psi {(}^{1}D_{2})=a\Phi {(}^{1}D_{2})+b\Phi {(}^{3}P_{2})\\ \Psi {(}^{3}P_{2})=-b\Phi {(}^{1}D_{2})+a\Phi {(}^{3}P_{2})\\ \Psi {(}^{3}P_{1})=\Phi {(}^{3}P_{1})\\ \Psi {(}^{3}P_{0})=c\Phi {(}^{3}P_{0})+d\Phi {(}^{1}S_{0})\\ \Psi {(}^{1}S_{0})=-d\Phi {(}^{3}P_{0})+c\Phi {(}^{1}S_{0}) \end{array}$$

We have used their line strength formulae, but the parameters have been determined in the manner used by Garstang [e.g., 42]. The electrostatic parameters E(^3^P), E(^1^D) and E(^1^S) (for *p^2^* and *p*^4^ configurations) or E(^4^S), E(^2^D), and E(^2^P) (for *p*^3^ configurations) and the spin-orbit parameter *ζ* have been treated as adjustable. They have been determined by fitting the theory [4, p. 268] to the observed energy levels by trial and error followed by one or more least squares adjustments. The energies were taken from [2] except for In [20] and Br II [19]. The adopted parameters are listed in tables 12, 17, and 21. The intermediate coupling wave functions are written in the form given by Shortley, Aller, Baker, and Menzel [27]:

for *p^2^* and *p*^4^ configurations, and

$$\begin{array}{l} \Psi {(}^{2}D_{5/2})=\Phi {(}^{2}D_{5/2})\\ \Psi {(}^{2}D_{3/2})=a\Phi {(}^{2}P_{3/2})+b\Phi {(}^{4}S_{3/2})+c\Phi {(}^{2}D_{3/2})\\ \Psi {(}^{4}S_{3/2})=a^{\prime }\Phi {(}^{2}P_{3/2})+b^{\prime }\Phi {(}^{4}S_{3/2})+c^{\prime }\Phi {(}^{2}D_{3/2})\\ \Psi {(}^{2}S_{3/2})=a^{\prime \prime }\Phi {(}^{2}P_{3/2})+b^{\prime \prime }\Phi {(}^{4}S_{3/2})+c^{\prime \prime }\Phi {(}^{2}D_{3/2})\\ \Psi {(}^{2}P_{½})=\Phi {(}^{2}P_{½}) \end{array}$$

for *p*^3^ configurations, where Φ and Ψ denote respectively the *LS*-coupling and intermediate coupling functions. The observed and calculated energies are listed in tables 13, 18, 22, 23, and 24, and the coefficients in the wave functions in tables 14 and 19. One check which can be applied in a few cases is to calculate the Landé *g*-values and compare them with observed values. This was done for Ge I, Sn I, and Pb I and the results are given in table 15. The general agreement of observed and calculated energy levels and Landé *g*-factors is very satisfactory, and lead us to think that the intermediate coupling theory provides a satisfactory representation of the atomic electron configurations. The radial integrals *s_g_* (defined as above with the 2/5 factor) needed in the formulae of [27] for electric quadrupole radiation were estimated as discussed below. Then the transition probabilities were calculated, and are given in tables 16, 20, and 25.

It should be mentioned that a number of authors have attempted fitting theory to observation for *p^n^* configurations, for example Te I, I II, and Xe III [20], Pb I [18, 43], Br II [19], Ge I [44], Sn I [45], and possibly others. Most assumed the Slater ratio between the term intervals, and thus had one fewer adjustable parameters than we use. The writer has given reasons [46] for prefering to treat the term intervals as unconnected by a Slater relation whenever this is feasible (as it is for *p^n^* configurations), and accordingly all the atoms have been treated in this way in the present work on *p^n^* configurations. Our use of an extra adjustable parameter results in a closer fit of theory and observation. In the only case where earlier work is comparable with ours [43] our results agree closely.

### 5.3. Estimation of *s_q_*

The most uncertain part of the calculations is the estimation of the radial integrals *s_q_.* For Ga I, Ge I, As I, Se I, and Br I (and for Kr I and Rb II, which were needed for extrapolation purposes) the selfconsistent field with exchange wave functions of Watson and Freeman [47] were used, and for Kr I as a check the wave function of Worsley [48]. For other 4*p^n^* configurations we assumed that the Rb II *minus* Kr I difference in *s_q_^−^*½ (linear in atomic number) should be added to the values of *s_q_^−^*½ for the neutral atoms to get the values for the corresponding ions, and by repeating the process values were obtained for doubly ionized atoms. Values of *s_q_* were collected for many atoms in the *3p^n^* [49, 52] and 2*p^n^* [42, 50, 51] configurations. These were used with the values for *Ap^n^* configurations to extrapolate *s_q_^−^*½ to the *5p^n^* and 6*p^n^* configurations. In these very rough calculations consideration was given to the variation of the effective quantum numbers derived from the energies and from the values of *s_q_* for 2*p^n^*, 3*p^n^*, and 4*p^n^* configurations. Some smoothing was applied to the estimates within the *5p^n^* and 6*p^n^* rows of atoms. The finally adopted values of *s_q_* are listed in table 26. The values of *s_q_*for the 4*p^n^* configurations are thought to be reasonably reliable; the values for the *5p^n^* and 6*p^n^* configurations are much less certain.

### 5.4. Accuracy of Results

The magnetic dipole transition probabilities do not depend on the radial integral *s_q_*, and they are therefore believed to be quite accurate, probably within 20 percent of the true values. It is very difficult to estimate the accuracy of our adopted values of *s_q_* and hence of the electric quadrupole transition probabilities. Jenkins and Mrozowski [33] showed that the line Pb I 6*p^2^* ^1^D_2_–^3^P_1_ λ7330 had 2.2 percent admixture of electric quadrupole radiation. According to our calculations the proportion is 3.3 percent, and 6.9 percent on combining the results of Gerjuoy [18] and Mrozowski [53]. This may suggest that the true *s_q_* are rather smaller than the ones we have adopted, the work of Jenkins and Mrozowski being believed to be fairly reliable. The calculated intensity ratios are *I* (4618): *1*(5313) = 9.0:1 and *I*(4659) : *1*(7330): *1*(9250) =0.0019:1:0.70, which may be compared with the observed values of Mrozowski [32] of 5.0:1 and 0.023:1:0.84. The discrepancy in *I*(4659) is not unduly disturbing because this line is rather sensitive to the parameters involved. The other calculated intensities are outside the probable errors given by Mrozowski, and the discrepancies cannot be explained by a reduction in *s_q_.* Probably there were some undetected errors in the experimental intensities. In Bi I, for λ6476 (^2^D_5/2_–^4^S_3/2_) the percentage of the total intensity due to electric quadrupole radiation is 20 percent according to our calculations; according to the observations of Mrozowski [38] the percentage did not exceed 15 percent (upper limit). This discrepancy could be explained if our *s_q_* were rather too large. The calculated percentage of electric quadrupole radiation in λ5640 (^2^P_3/2_–^2^D_5/2_) is about 30 percent; it does not exceed 10 percent for the other lines observed by Mrozowski. Our transition probabilities for Te I are in agreement with Niewodniczanski and Lipinski’s [40] comment (mentioned above) that λ5420 is the strongest line from ^1^S_0_. No other experimental results appear to be available. A new experimental determination of *s_q_* from intensity measurements for one or more ions would be of interest.

### 5.5. *d*^9^*s*^2^+*d*^10^*s* Configurations in Hg II

There is one other important group of forbidden lines in a heavy atom, those in Hg n. Dejardin [55] observed the line ^2^D_3/2_–^2^S_½_(λ1978), Paschen [56] observed ^2^D_5/2_–^2^S_½_(λ2815), and Naudé [57] observed ^2^D_3/2_–^2^D_5/2_(λ6647). These transitions occur within the configurations *d^9^s*^2^+*d*^10^*s.* Later observations were made by Sambursky [58] and Mrozowski [59]. Mrozowski showed that λ2815 is due to electric quadrupole radiation.

The line strengths of the transitions were obtained from the work of Shortley [7] for magnetic dipole radiation and from that of Garstang [16] for electric quadrupole radiation. As usual it is difficult to estimate the radial integrals involved. The results involve the integral

$$s_{q}(s,d)=\int_{0}^{\infty }r^{2}P(6s)P(5d)dr$$

where P(6*s*) is the radial wave function for the 6*s* electron in the 5*d*^9^6s^2^ configuration and P(5*d*) that for tlie 5*d* electron in the 5*d*^10^6*s* configuration. For a heavy atom relativistic wave functions should be used, and then the *d^10^* group subdivides into six *d* electrons and four $\bar{d}$ electrons, and the appropriate one must be used. The integral

$$s_{q}(d,\bar{d})=\int_{0}^{\infty }r^{2}P(5d)P(5\bar{d})dr$$

is also required for the transition ^2^D_3/2_–^2^D_5/2_. Wave functions for Hg II do not appear to be available, but relativistic sell-consistent field wave functions without exchange are available for neutral mercury, and we use the results of Mayers [60] for the 5*d*^10^6*s*^2^ configuration. From his wave functions it is found that *s_q_*(*s,d*) = –4.05, $s_{q}(s,\bar{d})=-3.53$, and $s_{q}(d,\bar{d})=+2.87$. Generally speaking the effect of exchange is to reduce the values of *s_q_*, and increased ionization has the same effect. For the purposes of making rough numerical estimates we have taken for the 5/2–1/2 transition *s_q_*(*s, d*) = −2.7, for the 3/2 – 1/2 transition $s_{q}(s,\bar{d})=-2.4$, and for the 3/2 —5/2 transition $s_{q}(d,\bar{d})=+2.0$. These values have been used in deriving the transition probabilities listed in table 27. It must be recognized, however, that these values of *s_q_* are only rough estimates. An accurate laboratory measurement of the ratio of λ1978 and λ6647 would be of exceptional interest.

## Figures and Tables

**Table 1:** Parameters for the *3d*^2^ configurations in *Ti III* and *Mn VI* (Units: *s_q_* in atomic units, others in cm^−1^)

Parameter	Ti III	Mn VI
		
E(^3^F)	243	974
E(^1^D)	8482	15446
E(^3^P)	10657	18274
E(^1^G)	14398	25502
E(^1^S)	(32881)	(57600)
ζ	118	465
*S_q_*	2.45	0.979

**Table 2:** Energy levels in the *3d*^2^ configurations in *Ti III* and *Mn VI* (Units: cm^−1^)

Level	Ti III			Mn VI		
	Obs.	Calc.	O–C	Obs.	Calc.	O–C
						
^3^F_2_	0	3	−3	0	10	−10
^3^F_3_	184	184	0	746	741	5
^3^F_4_	422	419	3	1669	1663	6
^1^D_2_	8473	8473	0	15336	15336	0
^3^P_0_	10536	10535	1	17782	17772	10
^3^P_1_	10604	10598	6	18057	18042	15
^3^P_2_	10721	10729	−8	18628	18652	−24
^1^G_4_	14399	14399	0	25511	25511	0
^1^S_0_	---------	(32885)	---------	---------	(57633)	---------

**Table 3:** Transition probabilities of [*Ti III*] and [*Mn VI*] (Units: sec^−1^)

Transition 3*d*^2^	Ti III		Mn VI	
	*A_m_*	*A_q_*	*A_m_*	*A_q_*
				
^3^F–^3^F 2–3	1.6×10^−4^	1.4×10^−12^	0.011	2.5×10^−10^
2–4	-----------	3.4×10^−12^	----------	4.8×10^−10^
3–4	2.7×10^−4^	4.3 ×10^−12^	0.016	6.0×10^−10^
^3^F–^4^D 2–2	0.0049	9.0×10^−6^	0.14	1.7×10^−4^
3–2	.0095	1.1×10^−5^	.23	2.3×10^−4^
4–2	------------	5.4×10^−5^	----------	9.0×10^−4^
^3^F–^3^P 2–0	---------	0.039	--------	0.087
2–1	2.7×10^−6^	.014	3.8×10^−4^	.031
3–1	-----------	.025	----------	.050
2–2	1.9×10^−5^	.0012	0.0035	.0028
3–2	7.3 ×10^−5^	.0079	.013	.017
4–2	----------	.027	----------	.050
^1^D–^3^P 2–0	----------	5.5×10^−8^	----------	1.6×10^−7^
2–1	0.0012	7.3×10^−8^	0.020	2.8×10^−7^
2–2	.0026	2.8×10^−9^	.060	2.6×10^−8^
^3^P–^3^P 0–1	5.4×10^−6^	----------	3.7×10^−4^	----------
0–2		1.1×10^−11^	----------	3.5×10^−9^
1–2	2.2 ×10^−5^	2.6×10^−12^	0.0024	1.1×10^−9^
^3^F–^1^G 2–4	----------	1.9 × 10^−5^	----------	2.5×10^−4^
3–4	0.0041	2.4×10^−7^	0.12	3.2×10^−6^
4–4	.0064	8.9×10^−6^	.17	1.1×10^−4^
^1^D–^1^G 2–4	----------	4.5×10^−4^	----------	0.0010
^3^P–^4^G 2–4	----------	3.2×10^−7^	----------	8.5×10^−6^
^3^F–^1^S 2–0	----------	0.0053	----------	0.063
^1^D–^1^S 2–0	----------	6.7	----------	16
^3^P–^1^S 1–0	0.098	----------	2.7	----------
2–0	----------	0.028	----------	0.55

**Table 4:** Radial integrals and correction factors to be applied to certain transition probabilities (Units: *s_q_* in atomic units)

Ion	s*_q_*	Correction factor^a^
		
Fe VII^b^	0.84	0.30
Cr IV	1.45	.144
Mn V	1.10	.185
Fe VI	.89	.24

a To be applied to electric quadrupole transition probabilities published by Pasternack [3].

b New wave junctions computed by S. J. Czyzak of Wright- Patterson Air Force Base [indicate 5*_q_*=0.80 forFe VII, leading to a correction factor 0.28.

**Table 5:** Electric quadrupole transition probabilities for *Cr II 3d^5 6^S* – *3d^4^4s ^6^D* (Units: sec^−1^)

Line	*A_a_*
	
^6^S_2½_ – ^6^D_½_	0.067
– ^6^D_1½_	.069
– ^6^D_2½_	.072
– ^6^D_3½_	.077
– ^6^D_4½_	.083

**Table 6:** Adopted parameters in *Ni I* (Units: *S_q_* in atomic units, others in cm^−1^)

*d*^8^*s*^2^ *ζ*	650
*α*	75
*A*	8181
*B*	1010
*C*	4179
*d^9^s F*_0_	1992
*G*_2_	1181
*ζ*′	604
*d*^10^ *E*_0_	14928
*s_q_*(3*d*^10^)	2.2
*s_q_*(3*d*^9^4*s*)	1.6
*s_q_*(3*d*^8^4*s*^2^)	1.1
*s_q_*(3*d*^10^–3*d*^9^4*s*)	−2.9
*s_q_*(3*d*^9^4*s*−3*d*^8^4*s*^2^)	−2.2

**Table 7:** Observed and calculated energy levels and Lande *g*-factors

Term	J	Energy levels		Landé *g*-factors		
		Observed	Calculated	Observed	LS-coupling	Calculated
						
		*cm*^−1^	*cm*^−1^			
3*d*^8^4*s*^2^ *a*^3^F	4	0	7	1.250	1.250	1.250
	3	1332^−1332^	1326^−1319^	1.083	1.083	1.083
	2	2217^−885^	2214^−888^	0.671	0.667	0.670
3*d*^9^4*s a*^3^D	3	205	207	1.332	1.333	1.333
	2	880^−675^	875^−668^	1.149	1.167	1.151
	1	1713^−833^	1717^−842^	0.497	0.500	0.500
3*d*^9^4*s a*^1^D	2	3410	3411	1.014	1.000	1.016
3*d*^8^4*s*^2^ *b*^1^D	2	13521	13491	1.143	1.000	1.128
3*d*^10^ *a*^1^ S	0	14729	14729	---------	----------	----------
3*d*^8^4*s*^2^ *a*^3^P	2	15610	15632	1.356	1.500	1.370
	1	15734^−124^	15726^−94^	1.497	1.500	1.500
	0	16017^−283^	15991^−265^	---------	---------	----------
3*d*^8^4*s*^2^ *a*^1^G	4	22102	22098	0.99	1.000	1.000
3*d*^8^4*s*^2 1^S	0	----------	51834	----------	----------	----------

**Table 8:** Transition probabilities of [*Ni I*] (Units: sec^−1^)

Transition		*A_m_*	*A_q_*
		
*a*^3^F–*a*^3^F	4–3	0.062	6.0×10^−9^
	4–2	---------------------	3.6×10^−9^
	3–2	.025	1.1× 10^−9^
*a*^3^F–*a*^3^D	4–3	---------------------	8.4×10^−11^
	3–3	---------------------	1.2×10^−7^
	2–3	---------------------	2.7×10^−7^
	4–2	---------------------	3.2×10^−8^
	3–2	---------------------	1.8×10^−9^
	2–2	---------------------	3.7×10^−7^
	3–1	---------------------	8.0×10^−10^
	2–1	---------------------	3.9×10^−9^
*a*^3^D-*a*^3^D	3–2	0.0070	4.1×10^−9^
	3–1	---------------------	3.0×10^−8^
	2–1	.021	1.7×10^−8^
*a*^3^F–*a*^4^D	4–2	---------------------	1.7×10^−6^
	3–2	---------------------	5.4×10^−7^
	2–2	---------------------	6.7×10^−9^
*a*^3^D–*a*^4^D	3–2	0.078	1.0× 10^−6^
	2–2	.0062	1.8×10^−7^
	1–2	.011	3.6×10^−^8
a^3^F–*b*^1^D	4–2	---------------------	0.0056
(1F)	3–2	0.39	7.6×10^−4^
	2–2	.17	2.0×10^−4^
*a*^3^D–*b*^1^D	3–2	---------------------	0.014
(4F)	2–2	---------------------	.017
	1–2	---------------------	4.5×10^−4^
*a*^1^D–*b*^1^D	2–2	---------------------	0.012
(6F)		---------------------	
*a*^3^F–*a*^1^S	2–0	---------------------	1.8×10^−4^
*a*^3^D–*a*^1^S	2–0	---------------------	0.068
*a*^3^D–*a*^1^S	2–0	---------------------	0.31
(7F)			
*b*^1^D–*a*^1^S	2–0	---------------------	2.9×10^−4^
*a*^3^F–*a*^3^P	4–2	---------------------	0.032
(2F)	3–2	0.15	.0056
	2–2	.039	3.98× 10^−4^
	3–1	---------------------	0.025
	2–1	.0022	.0090
	2–0	---------------------	.031
*a*^3^D-*a*^3^P	3–2	---------------------	0.074
(5F)	2–2	---------------------	.018
	1–2	---------------------	.0093
	3–1	---------------------	.092
	2–1	---------------------	.012
	1–1	---------------------	.053
	2–0	---------------------	.19
	1–0	---------------------	---------------------
*a*^1^D–*a*^3^P	2–2	---------------------	0.024
(8F)	2–1	---------------------	4.7×10-^−4^
	2–0	---------------------	1.1×10^−5^
*b*^1^D–*a*^3^P	2–2	0.072	1.9×10^−8^
	2–1	.063	6.9×10^−7^
	2–0	---------------------	1.2×10^−6^
*a*^1^S–*a*^3^P	0–2	---------------------	1.3× 10^−12^
	0–1	1.4×10^−4^	---------------------
*a*^3^P–*a*^3^P	2–1	3.2× 10^−5^	8.2×10^−13^
	2–0	0.0012	4.5×10^−10^
	1–0	---------------------	---------------------
*a*^3^F–*a*^1^G	4–4	0.32	2.2×10^−4^
(3F)	3–4	.16	3.8×10^−6^
	2–4	---------------------	3.0×10^−4^
*a*^3^D-*a*^1^G	3–4	---------------------	7.9×10^−4^
	2–4	---------------------	.080
*a*^1^D-*a*^1^G	2–4	---------------------	0.44
*b*^1^D-*a*^1^*G*	2–4	---------------------	4.1×10^−4^
*a*^3^P-*a*^1^G	2–4	---------------------	4.3×10^−5^
a^3^F-(^1^S)	2–0	---------------------	0.17
a^3^D-(^4^S)	2–0	---------------------	8.5
*a*^1^D-(^1^S)	2–0	---------------------	84
*b*^1^D-(^1^S)	2–0	---------------------	9.9
*a*^3^P-(^1^S)	2–0	---------------------	3.0
	1–0	5.4	---------------------

**Table 9:** Transition probabilities of [*Cu II*] (Units: sec^−1^)

Transition	*A_m_*	*A_q_*
		
3*d*^10^ ^1^S_0_–3d^9^4*s* ^3^D_2_	-------------	0.12
^1^S_0_– ^3^D_1_	-------------	-------------
3*d*^10^ ^1^S_0_–3*d*^9^4s ^1^D_2_	-------------	1.9
3*d*^9^4s ^3^D_3_–3*d*^9^4*s* ^3^D_2_	0.017	7.4× 10^−9^
^3^D_3_– ^3^D_1_	-------------	8.1 × 10^−8^
^3^D_2_– ^3^D_1_	.055	3.4×10^−8^
3*d*^9^4*s* ^3^D_3_–3d^9^4s ^1^D_2_	0.23	2.1 × 10^−6^
^3^D_2_– ^1^D_2_	.018	3.7 × 10^−7^
^3^D_1_– ^1^D_2_	.031	7.4× 10^−8^

**Table 10:** Transition probabilities for ${\text{np}\;}^{2}P_{\frac{3}{2}}\to^{2}P_{\frac{1}{2}}$ (Units: sec^−1^)

Ion	Config.	*A_m_*	*A_q_*
			
Ga I	4*p*	0.0050	1.0× 10^−6^
Ge II	4*p*	.049	3.1×10^−5^
As III	4*p*	.23	1.9×10^−4^
In I	5*p*	.097	2.7 × 10^−4^
Sn II	5*p*	.08	0.0035
Sb III	5*p*	2.5	.017
T1 I	6*p*	4.2	.11
Pb II	6*p*	25	1.3
Bi III	6*p*	80	5.9

**Table 11:** Transition probabilities for ${\text{np}}^{5}{\;}^{2}P_{\frac{1}{2}}\to^{2}P_{\frac{3}{2}}$ (Units: sec^−1^)

Ion	Config.	A*_m_*	A*_q_*
			
Br I	4*p*^5^	0. 89	8.3 ×10^−4^
Kr II	4*p*^5^	2. 8	0.0030
Bb III	4*p*^5^	7. 2	.0094
I I	5*p*^5^	7. 8	.055
Xe II	5*p*^5^	a21	.17
Cs III	5*p*^5^	48	.45
Rn II	6*p*^5^	a530	34

a These values have been given previously by Edlén [22].

**Table 12:** Parameters in *p^2^* configurations (Units: cm^−1^)

Parameter	Ge I	Sn I	Pb I	Bi II
	4*p^2^*	5*p^2^*	6*p^2^*	6*p*^2^
				
E(^3^P)	1023	2839	11538	19275
E(^1^D)	7050	8070	16930	25808
E(^1^S)	16263	16554	25205	36618
ζ	921.5	2247	7355	11789

**Table 13:** Observed and calculated energy levels in *p^2^* configurations (Units: cm^−1^)

Level	Ge I (4*p*^2^)			Sn I (5*p*^2^)			Pb I (6*p*^2^)			Bi II (6*p^2^*)		
	O	C	O–C	O	C	O–C	O	C	O–C	O	C	O–C
												
^3^P_0_	0	−3	3	0	−17	17	0	−94	94	0	−87	87
^3^P_1_	557	562	−5	1692	1715	−23	7819	7860	−41	13324	13380	−56
^3^P_2_	1410	1408	2	3428	3420	8	10650	10802	−152	17030	17147	−117
^1^D_2_	7125	7125	0	8613	8613	0	21458	21344	114	33936	33831	107
^1^S_0_	16367	16367	0	17163	17163	0	29467	29482	−15	44173	44190	−17

**Table 14:** Coefficients in intermediate coupling wave functions (*p^2^* and *p^4^* configurations)

Ion		*a*	*b*	*c*	*d*
				
Ge I	4*p*^2^	0.9934	0.1148	0.9968	0.0799
Sn I	5*p*^2^	.9463	.3232	.9821	.1883
Pb I	6*p*^2^	.7624	.6471	.9249	.3803
Bi II	6*p*^2^	.7206	.6933	.9106	.4133
Se I	4*p*^4^	.9910	−.1342	.9916	−.1297
Br II	4*p*^4^	.9863	−.1652	.9861	−.1663
Kr III	4*p*^4^	.9807	−.1953	.9784	−.2065
Te I	5*p*^4^	.9617	−.2743	.9471	−.3211
I II	5*p*^4^	.9523	−.3050	.9277	−.3732
Xe III	5*p*^4^	.9392	−.3435	.9039	−.4277
Po I	6*p*^4^	.8921	−.4519	.7495	−.6620

**Table 15:** Landé *g*-factors in *p^2^* configurations

Level	*LS* coupling	Ge I			Sn I			Pb I		
		Observed	Calculated	O–C	Observed	Calculated	O–C	Observed	Calculated	O–C
										
^3^P_1_	1.500	1.476	1.500	−0.024	1.502	1.500	0.002	1.501	1.500	0.001
^3^P_2_	1.500	1.514	1.493	.021	1.452	1.448	.004	1.269	1.291	−.022
^1^D_2_	1.000	.989	1.007	−.018	1.052	1.052	0	1.230	1.209	.021

**Table 16:** Transition probabilities for *p^2^* configurations (Units: sec^−1^)

Transition	Type	Ge I	Sn I	Pb I^a^	Bi II
					
^1^S_0_−^1^D_2_	*A_q_*	1.1	0.95	0.48	1.1
^1^S_0_−^3^P_2_	*A_q_*	0.068	0.57	10	47
^1^S_0_−^3^P_1_	*A_m_*	1.0	7.0	78	270
^1^D_2_–^3^P_2_	$\left\{ \begin{array}{l} A_{m}\\ A_{q} \end{array}\right.$	0.097	0.52	12	48
		.0010	.0061	0.60	3.7
^1^D_2_–^3^P_i_	$\left\{ \begin{array}{l} A_{m}\\ A_{q} \end{array}\right.$	0.050	0.46	14	56
		3.0×10^−4^	.0043	0.46	2.8
^1^D_2_–^3^P_0_	*A_q_*	3.0×10^−6^	8.1 × 10^−5^	0.0017	0.039
^3^P_2_–^3^P_1_	$\left\{ \begin{array}{l} A_{m}\\ A_{q} \end{array}\right.$	0.0082	0.062	0.18	0.35
		8.3 ×10^−7^	3.6×10^−5^	2.5×10^−4^	5.6 ×10^−4^
^3^P_2_–^3^P_0_	*A_q_*	4.6 ×10^−6^	5.9 ×10^−4^	0.21	1.6
^3^P_1_–^3^P_0_	*A_m_*	0.0031	0.083	7.3	35

a In substantial agreement with Gerjuoy [18], allowing for difference in *s_q_.*

**Table 17:** Parameters in *p^3^* configurations (Units: cm^−1^)

Parameter	As I	Sb I	Bi I
	4*p^3^*	5*p^3^*	6*p^3^*
			
E(^4^S)	116	672	7493
E(^2^D)	10919	9871	15348
E(^2^P)	18193	16407	21747
*ζ*	1441	3183	10159

**Table 18:** Observed and calculated energy levels in *p^3^* configurations (Units: cm^−1^)

Level	As I (4*p*^3^)			Sb I (5*p^3^*)			Bi I (6*p^3^*)		
	O	C	O–C	O	C	O–C	O	C	O–C
									
^4^S_3/2_	0	0	0	0	2	−2	0	−129	129
^2^D_3/2_	10593	10587	6	8512	8495	17	11419	11652	−233
^2^D_5/2_	10915	10919	−4	9854	9871	−17	15438	15348	90
^2^P_½_	18186	18193	−7	16396	16407	−11	21661	21747	−86
^2^P_3/2_	18648	18641	7	18465	18453	12	33165	33064	101

**Table 19:** Coefficients in intermediate coupling wave functions (*p^3^* configurations)

	As I	Sb I	Bi I
	4*p^3^*	5*p^3^*	6*p^3^*
			
*a*	−0.2021	−0.3572	−0.2466
*b*	−.0279	−.1453	−.6032
*c*	.9789	.9227	.7585
*a*′	−.0800	−.2053	−.5494
*b*′	.9968	.9759	.7318
*c*′	.0119	.0742	.4033
*a*″	.9761	.9113	.7984
*b*″	.0760	.1629	.3172
*c*″	.2038	.3781	.5118

**Table 20:** Transition probabilities for *p^3^* configurations (Units: sec^−1^)

Transition	Type	As I	Sb I	Bi I
				
^2^P_3/2_–^2^P_½_	$\left\{ \begin{array}{l} A_{m}\\ A_{q} \end{array}\right.$	8.4×10^−4^	0.065	8.6
		1.9×10^−9^	2.0×10^−5^	0.21
^2^P_3/2_–^2^D_5/2_	$\left\{ \begin{array}{l} A_{m}\\ A_{q} \end{array}\right.$	0.31	1.5	23
		.13	0.33	10
^2^P_3/2_–^2^D_3/2_	$\left\{ \begin{array}{l} A_{m}\\ A_{q} \end{array}\right.$	0.61	4.0	120
		.062	0.17	4.5
^2^P_½_–^2^D_5/2_	*A_q_*	0.059	.058	0.050
^2^P_½_–^2^D_3/2_	$\left\{ \begin{array}{l} A_{m}\\ A_{q} \end{array}\right.$	.32	1.1	1.2
		.10	0.19	0.51
^2^P_3/2_–^4^S_3/2_	$\left\{ \begin{array}{l} A_{m}\\ A_{q} \end{array}\right.$	1.6	5.2	7.3
		1.0×10^−4^	7.8×10^−4^	0.27
^2^P_½_–^4^S_3/2_	$\left\{ \begin{array}{l} A_{m}\\ A_{q} \end{array}\right.$	0.69	3.3	55
		.0012	0.048	6.2
^2^D_5/2_–^2^D_3/2_	$\left\{ \begin{array}{l} A_{m}\\ A_{q} \end{array}\right.$	3.4×10^−4^	.022	0.40
		4.7×10^−10^	3.2×10^−6^	4.0×10^−4^
^2^D_5/2_–^4^S_3/2_	$\left\{ \begin{array}{l} A_{m}\\ A_{q} \end{array}\right.$	0.0020	0.056	6.4
		.0033	.022	1.6
^2^D_3/2_–^4^S_3/2_	$\left\{ \begin{array}{l} A_{m}\\ A_{q} \end{array}\right.$	0.073	1.1	31
		.0019	0.0075	0.21

**Table 21:** Parameters in *p^4^* configurations (Units: cm^−1^)

Parameter	Se I	Br II	Kr III	Te I	I II	Xe III	Po I
	4*p*^4^	4*p*^4^	4*p*^4^	5*p*^4^	5*p*^4^	5*p*^4^	6*p*^1^
							
E(^3^P)	1075	1728	2549	2735	4133	5797	10611
E(^1^D)	9402	11759	14084	9765	12453	15083	17250
E(^1^S)	22112	27202	31890	21293	26310	32044	27295
*ζ*	1808	2785	3965	3954	5617	7906	12341

**Table 22:** Observed and calculated energy levels in *4p^4^* configurations (Units: cm^−1^)

Level	Se I			Br II			Kr III		
	O	C	O–C	O	C	O–C	O	C	O–C
									
^3^P_2_	0	−3	3	0	6	−6	0	8	−8
^3^P_1_	1989	1979	10	3136	3120	16	4548	4531	17
^3^P_0_	2534	2549	−15	3838	3849	−11	5313	5330	−17
^1^D_2_	9576	9576	0	12089	12089	0	14644	14643	1
^1^S_0_	22446	22446	0	27867	27866	1	33079	33074	5

**Table 23:** Observed and calculated energy levels in *5p^4^* configurations (Units: cm^−1^)

Level	Te I			I II			Xe III		
	O	C	O–C	O	C	O–C	O	C	O–C
									
^3^P_2_	0	−39	39	0	53	−53	0	−201	201
^3^P_1_	4751	4712	39	7087	6942	145	9795	9750	45
^3^P_0_	4707	4794	−87	6448	6555	−107	8131	8414	−283
^1^D_2_	10559	10562	−3	13727	13725	2	17100	17128	−28
^1^S_0_	23199	23188	11	29501	29505	−4	37398	37333	65

**Table 24:** Observed and calculated energy levels in *Po I* (*6p^4^*) (Units: cm^−1^)

Level	Observed	Calculated	O–C
			
^3^P_2_	0	13	−13
^3^P_1_	16831	16782	49
^3^P_0_	7514	7546	−32
^1^D_2_	21679	21671	8
^1^S_0_	42718	42710	8

**Table 25:** Transition probabilities for *p^4^* configurations (Units: sec^−1^)

Transition	Type	Se I	Br II	Kr III	Te I	I II	Xe III	Po I
								
^1^S_0_–^1^D_2_	*A_q_*	2.3	4.0	4.5	3.1	5.5	13	42
^1^S_0_–^3^P_2_	*A_q_*	0.18	0.43	0.69	0.79	1.6	3.9	4. 1
^1^S_0_–^3^P_1_	*A_m_*	7.7	a23	53	37	b84	210	410
^1^D_2_–^3^P_2_	$\left\{ \begin{array}{l} A_{m}\\ A_{q} \end{array}\right.$	0.62	a1.9	c4.7	3.3	b8.8	d 21	66
		.0074	0.022	0.043	0.077	0.21	0.52	9.3
^1^D_2_–^3^P_1_	$\left\{ \begin{array}{l} A_{m}\\ A_{q} \end{array}\right.$	.11	.26	c 0.53	.20	.36	c.62	0.31
		3.5×10^−4^	7.2× 10^−4^	.0010	6.0× 10^−4^	8.6×10^−4^	.0012	9.4× 10^−4^
^1^D_2_–^3^P_0_	*A_q_*	9.2×10^−5^	2.1× 10^−4^	3.7× 10^−4^	4.7×10^−4^	0.0012	.0031	0.31
e3P_0_–^3^P_1_	*A_m_*	0.0085	0.018	0.023	1.4× 10^−6^	.0040	.067	8.1
^3^P_0_–^3^P_2_	*A_q_*	1.8×10^−4^	8.7× 10^−4^	.0025	0.0073	.022	.052	0.15
^3^P_1_–^3^P_2_	$\left\{ \begin{array}{l} A_{m}\\ A_{q} \end{array}\right.$	0.17	0.67	2.0	2.2	7.2	d19	85
		3.9×10^−5^	2.2×10^−4^	7.6×10^−4^	*0.*0044	0.020	0.068	3.1

a Given by Martin and Tech [19] with whom we agree.

b Given by Martin and Corliss [20], who obtained 99, 9.1, respectively.

c Given by Osterbrock [21] with whom we agree.

d Given by Edlén [22] and Osterbrock [21], with whom we agree.

e ^3^P_0_→^3^P_1_ in Se I, Br II and Kr III, ^3^P_1_→^3^P_0_ in Te I, I II, Xe III, and Po I.

**Table 26:** Adopted values of $s_{q}=\frac{2}{5}\bar{r^{2}}$ for configurations^a^ (*s_q_* in atomic units)

		*p*	*p*^2^	*p*^3^	*p*^4^	*p*^5^
					
4	I	5.54	3.84	2.95	2.48	2.09
	II	3.58			1.9	1.58
	III	2.51			1.4	1.24
5	I	6.0	4.5	3.8	3.1	2.8
	II	4.3			2.4	2.2
	III	3.2			2.0	1.8
6	I	5.3	4.4	4.0	3.7	
	II	4.1	3.5			2.1
	III	3.3				

a 4, 5, 6 are the principal quantum numbers, I, II, III are spectrum numbers; e.g., 4*p*^5^ Kr II has *s_q_*=1.58. Atomic units used.

**Table 27:** Transition probabilities in [*Hg II*] (Units: sec^−1^)

Transition	*A_m_*	*A_q_*
		
5*d*^9^6*s*^2 2^D_3/2_–5*d*^9^6*s*^2^ ^2^D_5/2_	54	0.029
5*d*^9^6*s*^2 2^D_3__/2_–5*d*^10^6*s* ^2^S_1/2_	----------	42
5*d*^9^6*s*^2 2^D_5__/2_–5*d*^10^6*s* ^2^S_1/2_	----------	9.5

**Table 28:** Electric quadrupole multiplet strengths

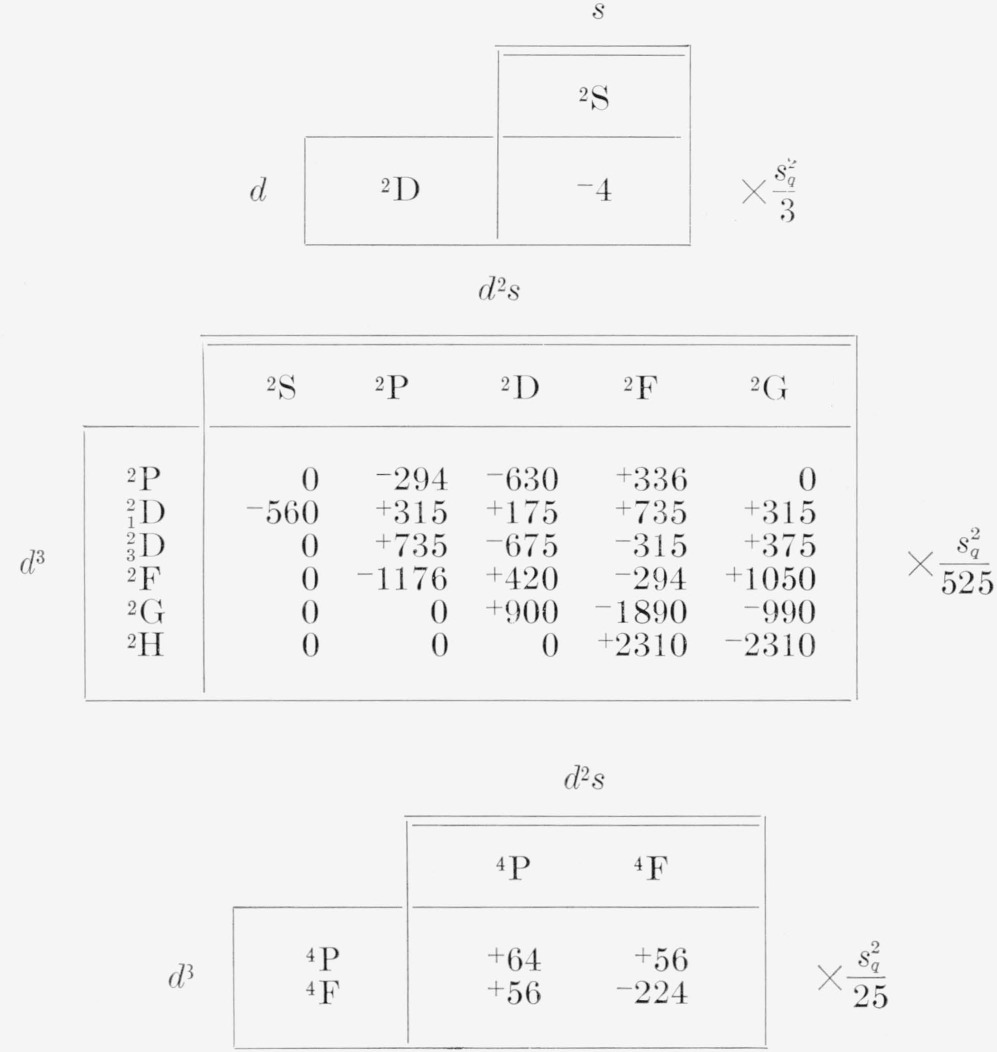
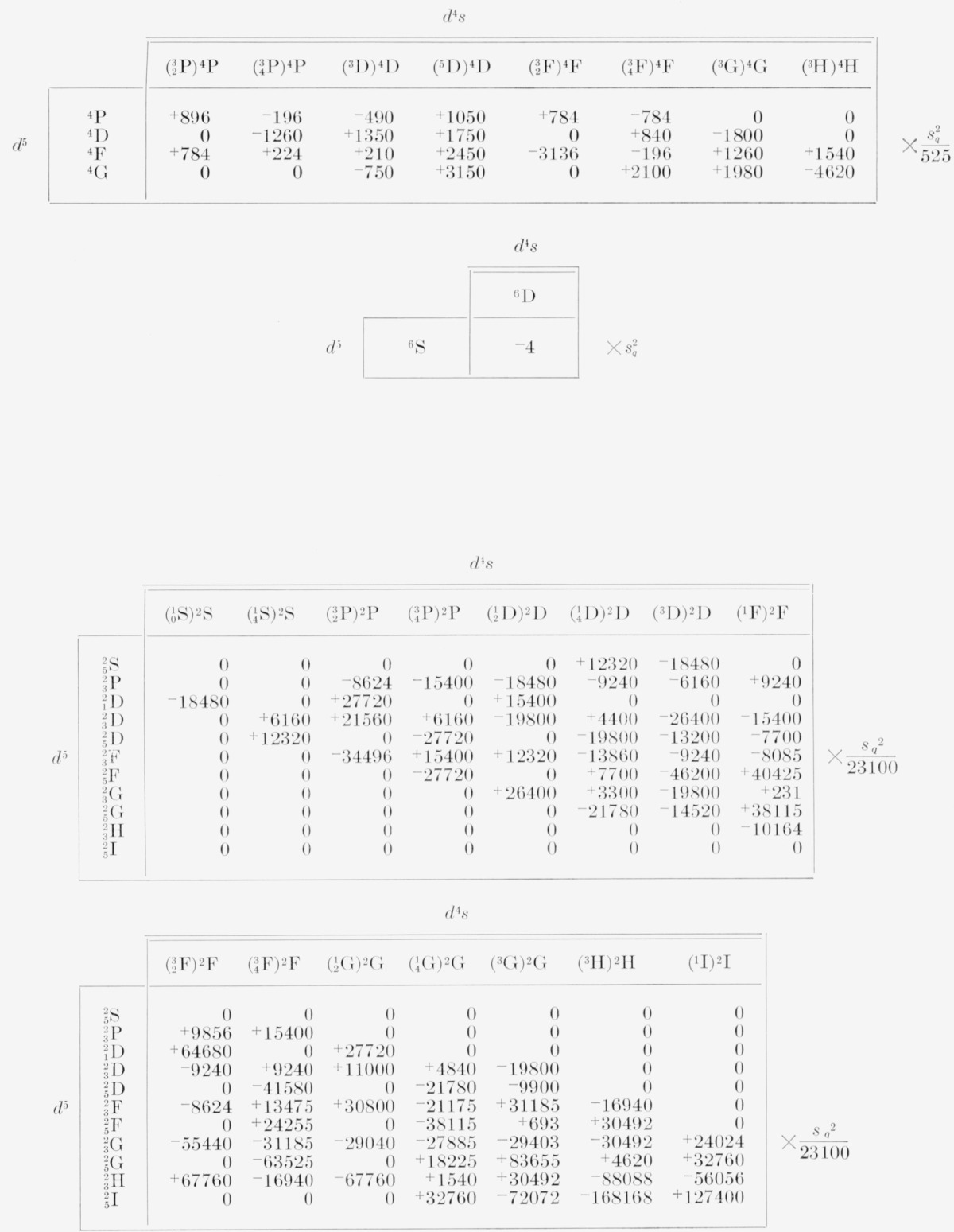

## Footnotes

2 Figures in brackets indicate the literature references on page 71.

## 7. Appendix. Electric Quadrupole Multiplet Strengths for the Transition Arrays *d^n^*–*d^n^*^−^*^1^s*

In an earlier paper Gars tang [16] showed how to calculate electric quadrupole line strengths for many transitions involving *s* and *d* electrons. Among these were the transitions *d^n^*–*d^n^^−^*^1^*s.* Detailed results were given for *d*^9^–*d*^8^*s* and *d^7^–d*^6^*s.* It was also shown that *S_q_(d^n^αSL–d^n−^*^1^(*α*_1_*S*_1_*L*_1_*)sSL*_1_) = *S_q_*(*d*^11^*^−n^ a*_1_*S*_1_*L*_1_–*d*^10^*^−n^*(*αSL)sS*_1_*L*) so that values for the arrays *d*^11^*^−n^–d*^10^*^−n^s* can be obtained from those for *d^n^–d^n−^*^1^*s.* The phases of the matrix elements for the former array are ${\left(-1\right)}^{S+S_{1}+L+L_{1}-½}$ times those of *d^n^–d^n−^*^1^*s;* an extra (–1) has to be introduced for *n*=6 if *α*_1_*S*_1_*L*_1_ has seniority 3. Thus the transitions *d^2^–ds* and *d*^4^–*d*^3^*s* need not be listed. We list here results for *n*= 1, 3, and 5. The tabulated quantities are *S_g_*(*αSL, a*′*SL*′), as multiples of $s_{q}^{2}$ where

$$s_{q}=\int_{0}^{\infty }r^{2}P(d)P(s)dr$$

involves the radial wave functions of the *d* electron in the *d^n^* configuration and the *s* electron in the *d^n−^*^1^*s* configuration. As superscript prefixes to the strengths there have been inserted the signs of the square roots of the strengths (i.e., of the matrix elements *(αSL*‖*T*^(2)^‖*a*′*SL*′)) required in determining the phases for intermediate coupling calculations. Racah’s seniority numbers are given as subscripts to the term symbols. The strengths of individual lines may be obtained (in *LS*-coupling) in cases where there is only one term of the same kind as each of the initial and final terms of the desired transition by a direct application of [8, eqs (3), (5), and (6)]. In cases where there are more than one term of the same kind in either or both of the initial and final states, much more elaborate calculations must be performed, as for example for Fe II [12].

## References

[1] Garstang RH, Bates DR (1962). Atomic and Molecular Processes.

[2] Moore CE (1949). Atomic energy levels.

[3] Pasternack S (1940). Astrophys J.

[4] Condon EU, Shortley GH (1951). The Theory of Atomic Spectra.

[5] Racah G (1942). Phys Rev.

[6] Trees RE (1952). Phys Rev.

[7] II G (1940). Shortley, Phys Rev.

[8] Garstang RH (1957). Proc Cambridge Phil Soc.

[9] Watson RE (1959). Tech Report 12, Solid State and Molecular Theory Group.

[10] Bowen IS (1955). Astrophys J.

[11] Thackeray AD (1962). Monthly Notices Roy Astron Soc.

[12] Garstang RH (1962). Monthly Notices Roy Astron Soc.

[13] Garstang RH (1958). Monthly Notices Roy Astron Soc.

[14] Racah G (1943). Phys Rev.

[15] Racah G, Shadmi Y (1959). Bull Res Council Israel.

[16] Garstang RH (1958). Proc Cambridge Phil Soc.

[17] Thackeray AD (1953). Monthly Notices Roy Astron Soc.

[18] Gerjuoy E (1941). Phys Rev.

[19] Martin WC, Tech JL (1961). J Opt Soc Am.

[20] Martin WC, Corliss CH (1960). J Res NBS.

[21] Osterbrock DE (1951). Astrophys J.

[22] Edlen B (1944). Phvs Rev.

[23] Walters FM (1922). BS Sei Pap.

[24] Cole CD (1961). Bull Am Phys Soc.

[25] Eshbach FE, Fisher RA (1954). J Opt Soc Am.

[26] Kiess CC, Corliss CH (1959). J Res NBS.

[27] Shortley GH, Aller LH, Baker JG, Menzel DH (1941). Astrophys J.

[28] Niewodniczanski H (1934). Acta Phys Polonica.

[29] Gieseler H, Grotrian W (1925). Zeit Physik.

[30] Sur NK (1926). Phil Mag.

[31] Niewodniczanski H (1933). Acta Phys Polonica.

[32] Mrozowski S (1940). Phys Rev.

[33] Jenkins FA, Mrozowski S (1941). Phys Rev.

[34] Cole CD, Mrozowski S (1954). Phys Rev.

[35] Cole CD (1960). Bull Am Phvs Soc.

[36] Hults M, Mrozowski S (1952). Phys Rev.

[37] Toshniwal GR (1927). Phil Mag.

[38] Mrozowski S (1946). Phys Rev.

[39] Ruedv JE, Gibbs RC (1934). Phys Rev.

[40] Niewodnicanski H, Lipinski F (1938). Nature.

[41] Mrozowski S (1956). J Opt Soc Am.

[42] Garstang RH (1951). Monthly Notices Roy Astron Soc.

[43] Helliwell TM (1961). Astrophys J.

[44] van den Bosch JC, Klinkenberg PFA (1941). Proc Ned Akad Wetens.

[45] Goudsmit S (1930). Phys Rev.

[46] Garstang RH (1956). Proc Cambridge Phil Soc.

[47] Watson RE, Freeman AJ (1961). Phys Rev.

[48] Worsley BH (1958). Proc Roy Soc.

[49] Czyzak SJ (1962). Astrophys J Supp.

[50] Garstang RH (1952). Astrophys J.

[51] Worsley BH (1958). Can J Phys.

[52] Hartree DR, Hartree W (1938). Proc Roy Soc.

[53] Mrozowski S (1944). Reviews Mod Phys.

[54] Joy AH, Swings P (1945). Astrophys J.

[55] Déjardin G (1927). Ann Phys.

[56] Paschen F (1928). Ber Preuss Akad Wiss.

[57] Naudé SM (1929). Ann Physik.

[58] Samburskv S (1932). Zeit Physik.

[59] Mrozowski S (1940). Phys Rev.

[60] Mayers DF (1957). Proc Roy Soc.

[61] Joy AH (1961). Astrophys J.

